# Correction: The changing meaning of “no” in Canadian sex work

**DOI:** 10.1371/journal.pone.0306019

**Published:** 2024-06-18

**Authors:** Lynn Kennedy

The images for Figs 1 and 2 are incorrectly switched. The image that appears as Fig 1 should be Fig 2, and the image that appears as Fig 2 should be Fig 1. The figure captions appear in the correct order. Please see the correct Fig 1 and Fig 2 here.

**Fig 1:**
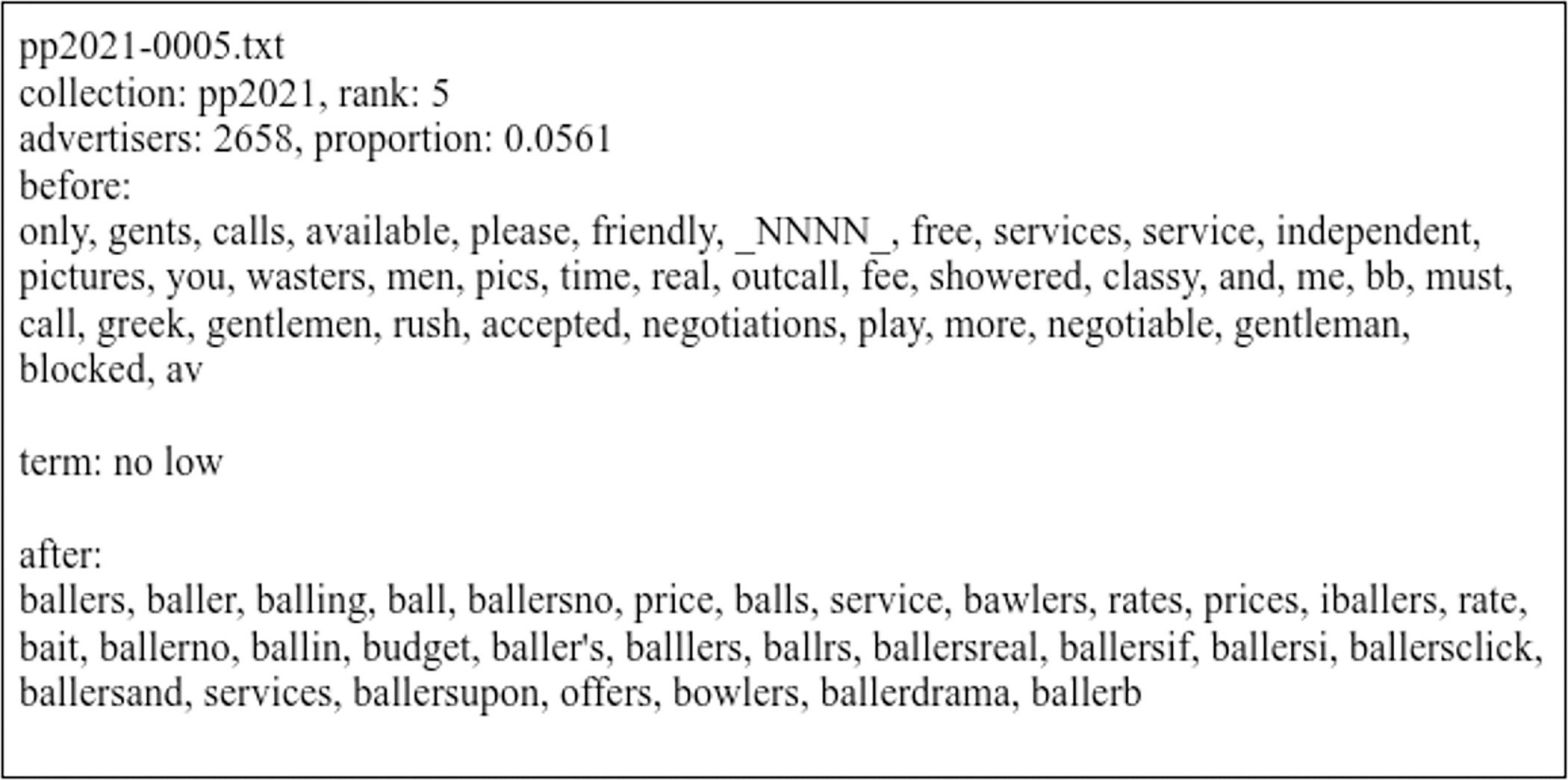
Example coding file for the bigram “no low” for the 2021–2022 collection. “Before” and “after” words are terms that preceded and followed the bigram ordered in descending order by advertiser frequency. “_NNNN_” replaces a four-digit number in the anonymized data.

**Fig 2:**
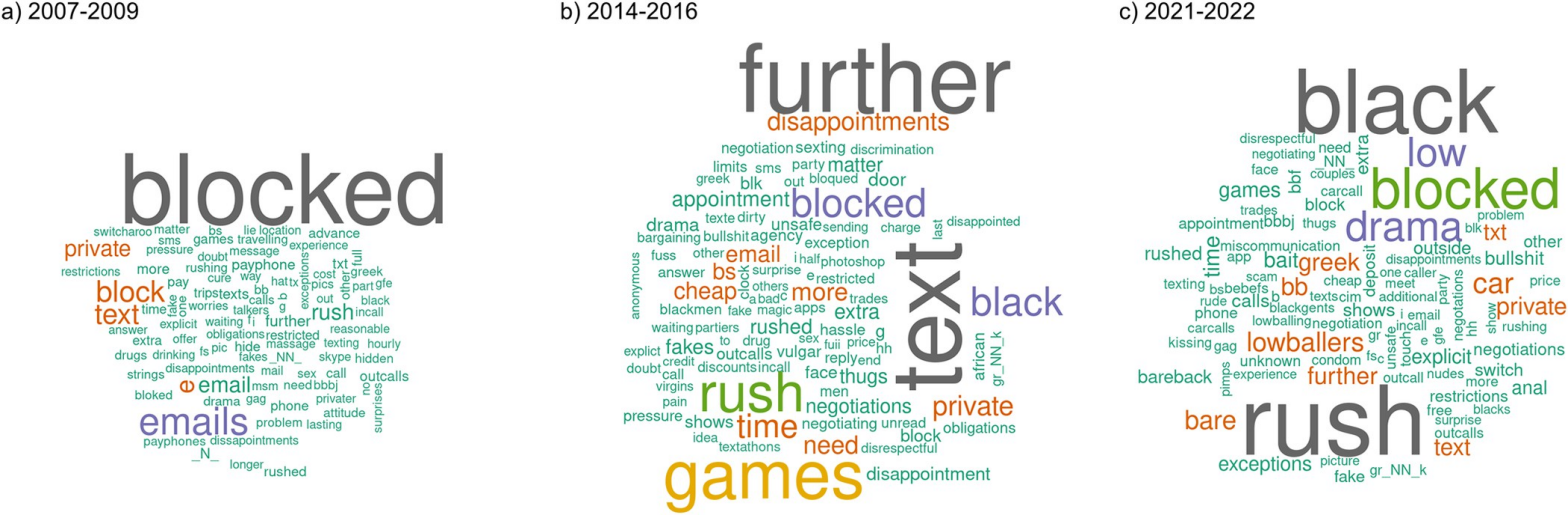
Top 100 terms following the word “no” by time period based on term frequency. Larger words represent more frequently used terms.
