# Supplementary material for: The changing meaning of “no” in Canadian sex work
Source: PLoS One. 2024 Apr 4;19(4):e0301600. doi: 10.1371/journal.pone.0301600 (PMC10994304; doi:10.1371/journal.pone.0301600)
Supplement: S1 Appendix — https://osf.io/ys5ed. (DOCX) [file pone.0301600.s001.docx]

Craigslist.com/ers in Vancouver 2007-2009

Fig 1 illustrates the rapid adoption of the craigslist.com erotic services (ers) section between March 1, 2007 and March 31, 2009. In this time the number of workers was estimated to be 13497 (95% CI 13319 - 13684). Monthly mean 1579 (SD 612) workers were estimated to be active each month increasing from an estimated 488 workers (95 % CI 484 - 492) in March 2007 to 2658 workers (95 % CI 2641 - 2676) in March 2009. Estimates were created as described in (Kennedy, 2022). Source data can be found here: <https://osf.io/mbdyg> .

**Fig 1:Ads and advertisers on craigslist.com erotic services section March 2007 to March 2009.** Note that data was missing for part of the month of June, 2008.


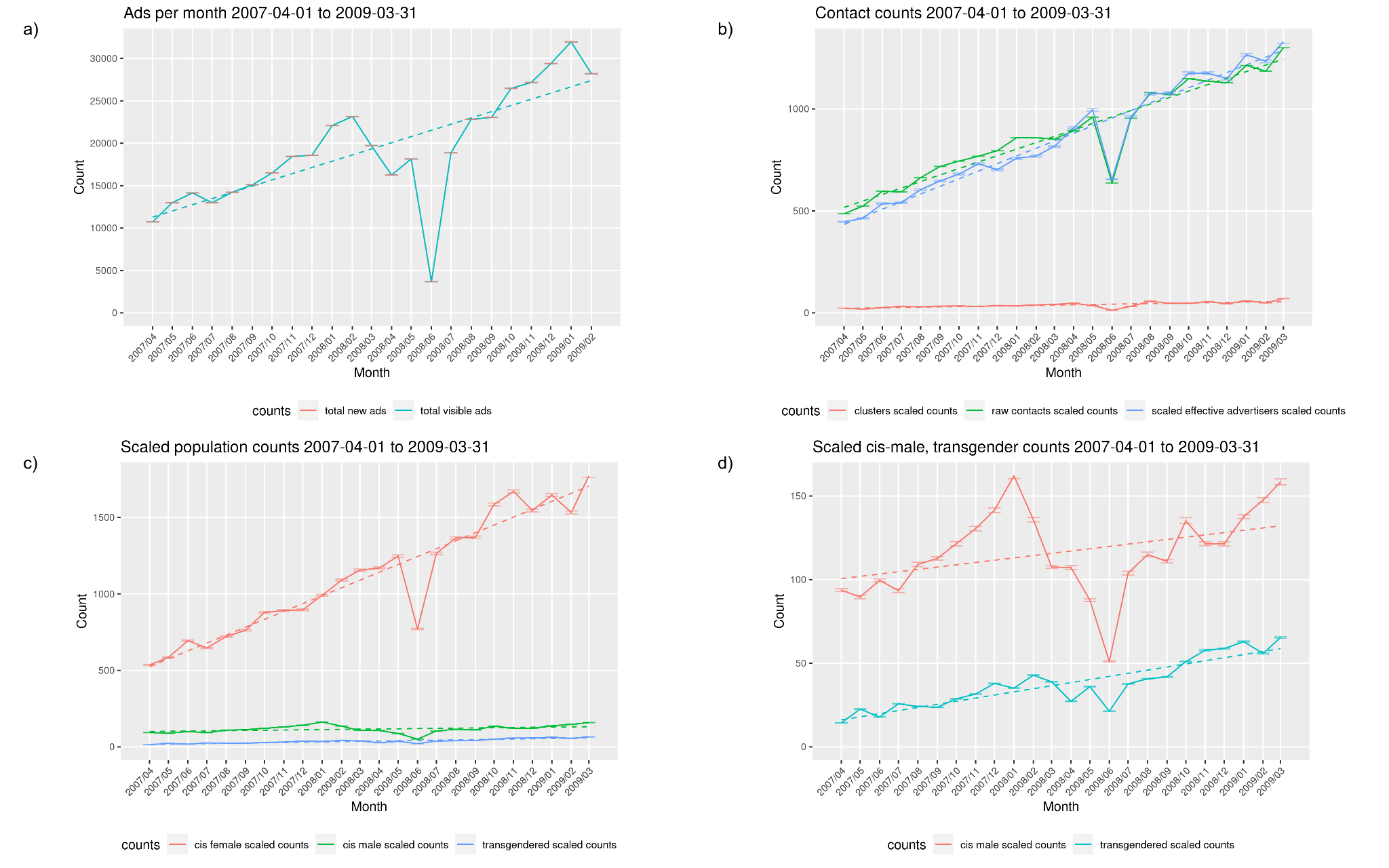


Kennedy, L. (2022). The silent majority: The typical Canadian sex worker may not be who we think. *PloS One*, *17*(11), e0277550–e0277550.
