# Supplementary material for: The changing meaning of “no” in Canadian sex work
Source: PLoS One. 2024 Apr 4;19(4):e0301600. doi: 10.1371/journal.pone.0301600 (PMC10994304; doi:10.1371/journal.pone.0301600)
Supplement: S2 Appendix — https://osf.io/wdj3t. (DOCX) [file pone.0301600.s002.docx]

Effect of Weighting on Probability Estimates

# Introduction

To better understand the effect of advertisers using multiple identifiers, an exploratory analysis was undertaken where the effect of short-term advertisers was reduced. In a previous study, those advertising for short periods of time appeared to be much more likely to be using multiple identifiers based on image sharing data (Kennedy 2022). To see what effect discounting short term advertisers would have, weighted probability estimates are compared with the original raw count estimates.

The measure used in this study is the estimated probability of association between the variables of time period, region, gender and ethnicity and one of the themes *peace of mind*, *communication*, *service restrictions*, *client behavior* and *client race* (e.g. p(*client behavior*=True | gender=*male*)). Subgroups of advertisers associated with a theme are counted, and this count is divided by the count of all advertisers associated with that subgroup. Weighted probability estimates reduce the original raw counts using weights, where an advertiser can count as less than one if they advertised for less than the study period.

Weights were generated based on the formula described in (Kennedy 2022):

$P(a unique) =\frac{1}{1 + New Contacts} = \frac{1}{1 + \left( \frac{Period}{Days(a)} - 1 \right)\cdot R_{idchange}}$ (1)

Where *P(a unique)* is the probability estimate that any given advertiser was uniquely identified by that contact, used in this analysis as the weight for that advertiser. *Period* represents the total length of the measurement period, *Days(a)* was the measured number of days the advertiser was active based on the date of the first and last ad, ranging from 1 ≤ *Days(a)* ≤ *Period*, and *R_idchange_* was the measured rate at which advertisers changed contacts.

For the purposes of this analysis, *R_idchange_* is estimated to be 0.0223 contacts per day based on (Kennedy 2022). *Period* was set at 730 days for the 2007-2009 and 2014-2016 collections and 365 days for the 2021-2022 collection. Figure 1 shows the relationship between *P(a unique)* as a function of *Days(a)* where *Period* is set to 730.

**Figure 1**: *P(a unique)* as a function of *Days(a)*.


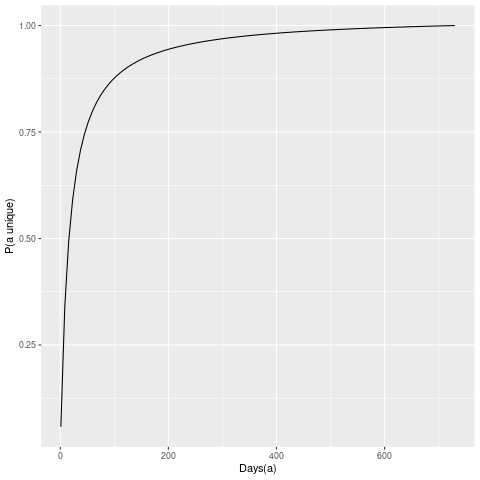


In this weighting scheme, advertisers who were active for the whole period were given a weight of 1. However, an advertiser who had only advertised for a single day in a 730-day period would be given a weight of 0.0579. Weights were summed to generate the weighted probability estimates.

# Comparing probabilities by theme

The weighted and raw count probability estimates of the associations between themes and variables were compared. The weighted estimates were not significantly less than the raw count estimates based on tests using the R *prop.test* function (R Core Team 2021) and in most cases (119/129) were greater than the raw count estimates.

Some variable rankings for the *client race*, *service restrictions*, *communication* and *peace of mind* themes were changed in the weighted estimates. For the *client race* theme, the rankings for the regions of *Quebec* and *East* swapped places. This was also the case for worker ethnicities *Indo Canadian* and *Black*. For the *service restrictions* theme, worker ethnicities *Asian* and *Middle Eastern* swapped ranks. For the *communication* theme the regions *Unknown* and *Quebec* swapped ranks as did *British Columbia* and *Ontario*; and the raw count estimate worker ethnicity ranks (from smallest to largest) *Asian Canadian*, *Native*, *Middle Eastern* were reordered in the weighted estimates to *Native*, *Middle Eastern*, *Asian Canadian*. Lastly, for the *peace of mind* theme, *British Columbia* was ranked sixth in the raw count estimates and third in the weighted estimates and the ranks of the worker ethnicities *Black* and *Hispanic* were swapped. These differences do not substantially affect the overall results of the study, as the weighted estimates are similar where changes in rank occur.

Figures 2 through 6 show side by side comparisons of the distributions for the themes *client race*, *client behavior*, *service restrictions*, *communication* and *peace of mind* respectively.

**Figure 2**: *Client race* comparison between unweighted and weighted probabilities.


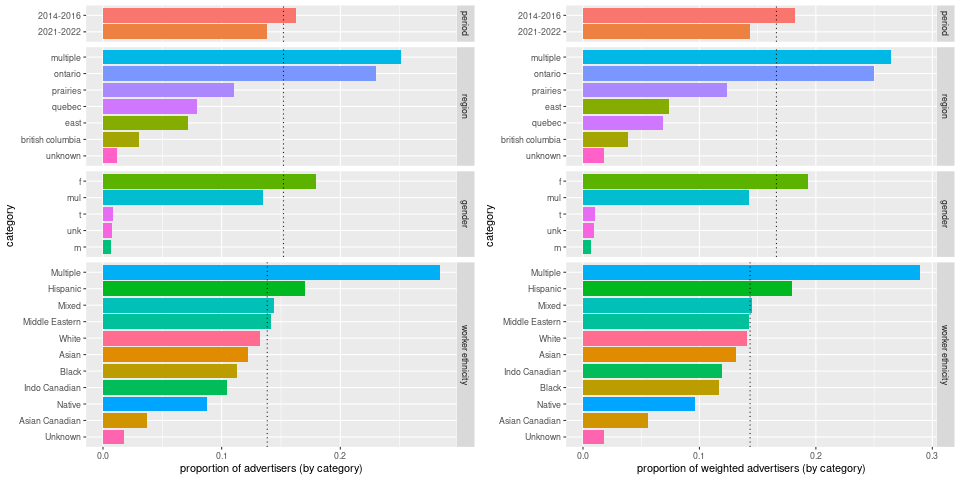


**Figure 3:** *Client behavior* comparison between unweighted and weighted probabilities.


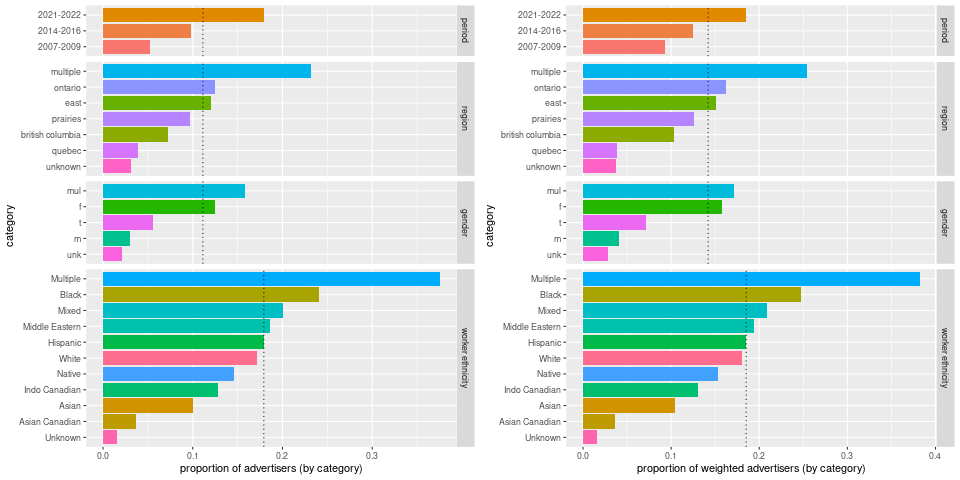


**Figure 4:** *Service restrictions* comparison between unweighted and weighted probabilities.


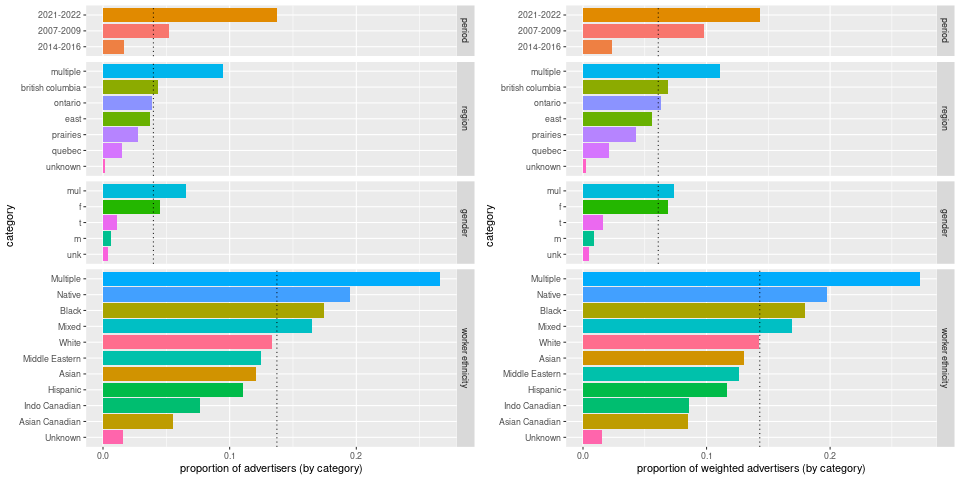


**Figure 5:** *Communication* comparison between unweighted and weighted probabilities.


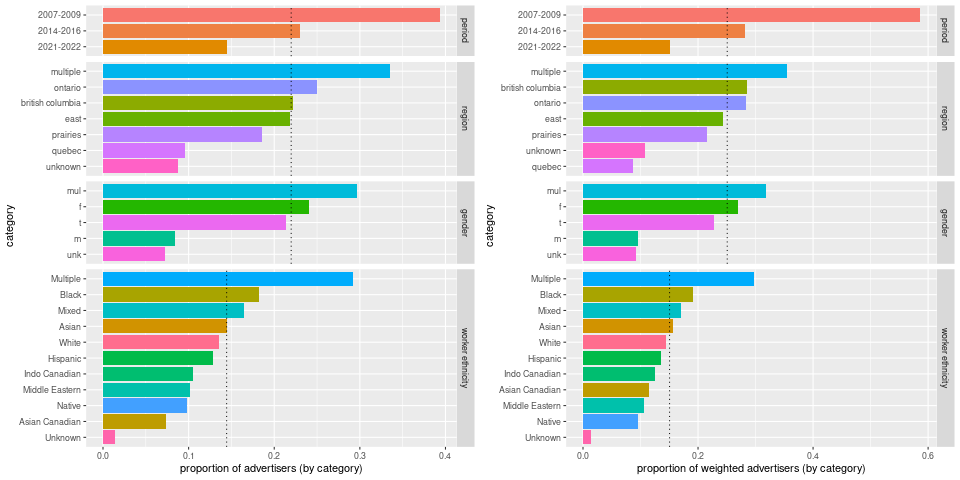


**Figure 6:** *Peace of mind* comparison between unweighted and weighted probabilities.


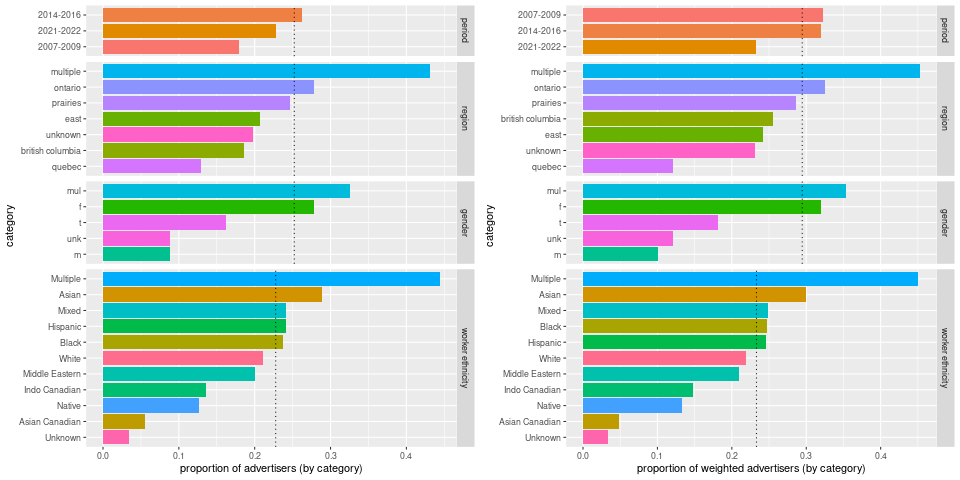


# Bibliography

Kennedy, Lynn. 2022. “The Silent Majority: The Typical Canadian Sex Worker May Not Be Who We Think.” *PloS One* 17(11):e0277550–e0277550.

R Core Team. 2021. *R: A Language and Environment for Statistical Computing*. Vienna, Austria: R Foundation for Statistical Computing.
