# Supplementary figures and images for: The changing meaning of “no” in Canadian sex work
Source: PLoS One. 2024 Apr 4;19(4):e0301600. doi: 10.1371/journal.pone.0301600 (PMC10994304; doi:10.1371/journal.pone.0301600)

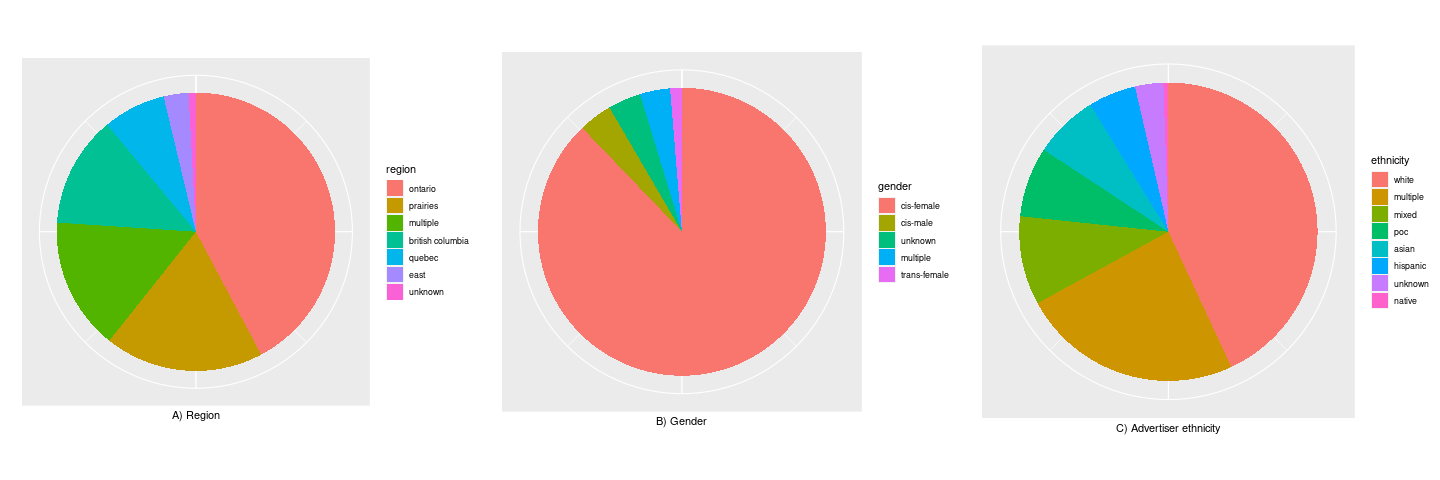

Supplement: S1 Fig — https://osf.io/8k234. (PNG) [file pone.0301600.s007.png]
